# Specialist versus Primary Care Prostate Cancer Follow-Up: A Process Evaluation of a Randomized Controlled Trial

**DOI:** 10.3390/cancers14133166

**Published:** 2022-06-28

**Authors:** Barbara M. Wollersheim, Kristel M. van Asselt, Floris J. Pos, Emine Akdemir, Shifra Crouse, Henk G. van der Poel, Neil K. Aaronson, Lonneke V. van de Poll-Franse, Annelies H. Boekhout

**Affiliations:** 1 Division of Psychosocial Research and Epidemiology, The Netherlands Cancer Institute, Antoni van Leeuwenhoek Hospital, Plesmanlaan 121, 1066 CX Amsterdam, The Netherlands; b.wollersheim@nki.nl (B.M.W.); e.akdemir@nki.nl (E.A.); shifracrouse1996@gmail.com (S.C.); n.aaronson@nki.nl (N.K.A.); l.vd.poll@nki.nl (L.V.v.d.P.-F.); 2 Department of General Practice, Amsterdam UMC, Location AMC, University of Amsterdam, Meibergdreef 9, 1105 AZ Amsterdam, The Netherlands; k.m.vanasselt@amsterdamumc.nl; 3 Department of Radiotherapy, The Netherlands Cancer Institute, Antoni van Leeuwenhoek Hospital, Plesmanlaan 121, 1066 CX Amsterdam, The Netherlands; f.pos@nki.nl; 4 Department of Urology, The Netherlands Cancer Institute, Antoni van Leeuwenhoek Hospital, Plesmanlaan 121, 1066 CX Amsterdam, The Netherlands; h.vd.poel@nki.nl; 5 Department of Research, Netherlands Comprehensive Cancer Organization (IKNL), Godebaldkwartier 419, 3511 DT Utrecht, The Netherlands; 6 Department of Medical and Clinical Psychology, CoRPS—Center of Research on Psychology in Somatic Diseases, Tilburg University, Warandelaan 2, 5037 AB Tilburg, The Netherlands

**Keywords:** prostate cancer survivors, follow-up care, primary health care, general practice, process evaluation, Consolidated Framework for Implementation Research

## Abstract

**Simple Summary:**

Worldwide, there is an increased focus on reorganizing prostate cancer survivorship care. In this study, we describe for the first time a process evaluation as part of a randomized controlled trial that is currently comparing the effectiveness of specialist- versus primary care-based prostate cancer follow-up. We found that within an RCT context, 67% patients and their GPs were willing to receive/provide primary care-based follow-up. Patients who received primary care-based follow-up care experienced this to be more personal, efficient, and sustainable. However, patients, GPs, and specialists also indicated several challenges that are described in this study and should be addressed to enable a smooth transition of prostate cancer follow-up to primary care.

**Abstract:**

*Background:* A randomized controlled trial (RCT) is currently comparing the effectiveness of specialist- versus primary care-based prostate cancer follow-up. This process evaluation assesses the reach and identified constructs for the implementation of primary care-based follow-up. *Methods:* A mixed-methods approach is used to assess the reach and the implementation through the Consolidated Framework for Implementation Research. We use quantitative data to evaluate the reach of the RCT and qualitative data (interviews) to indicate the perspectives of patients (*n* = 15), general practitioners (GPs) (*n* = 10), and specialists (*n* = 8). Thematic analysis is used to analyze the interview transcripts. *Results:* In total, we reached 402 (67%) patients from 12 hospitals and randomized them to specialist- (*n* = 201) or to primary care-based (*n* = 201) follow-up. From the interviews, we identify several advantages of primary care- versus specialist-based follow-up: it is closer to home, more accessible, and the relationship is more personal. Nevertheless, participants also identified challenges: guidelines should be implemented, communication and collaboration between primary and secondary care should be improved, quality indicators should be collected, and GPs should be compensated. *Conclusion:* Within an RCT context, 402 (67%) patients and their GPs were willing to receive/provide primary care-based follow-up. If the RCT shows that primary care is equally as effective as specialist-based follow-up, the challenges identified in this study need to be addressed to enable a smooth transition of prostate cancer follow-up to primary care.

## 1. Introduction

Prostate cancer follow-up care is in need of a more sustainable follow-up care model because of the increasing number of prostate cancer survivors and the demands to improve its quality and efficiency [1,2]. Currently, in most Western countries, prostate cancer survivors are included in a hospital-based follow-up care program [3]. Different models of follow-up care for (prostate) cancer patients have been proposed [4]; this could be risk-based on clinical outcomes [5], including shared-care models and primary care-based follow-up care models. Currently, we are conducting a prospective, randomized, multicenter study (PROSPEC trial) to compare the (cost-)effectiveness of specialist- (usual care) versus primary care-based (intervention) follow-up of patients who have completed their primary treatment for localized prostate cancer [6].

When investigating the efficacy of such a potential shift in the organization of care, it is important to evaluate a number of relevant process outcomes [7,8]. Reorganizing this routine demands close collaboration between primary and secondary care providers, and it asks for change in the behavior of patients, clinicians, and in the organization of care [9]. To date, most studies have focused on cancer survivors’ preferences for cancer follow-up care [10,11,12,13] and general practitioners’ (GPs) willingness to provide cancer follow-up care [14,15,16]. Since most of these studies were conducted in a setting where specialist-based follow-up was current practice, it is important to investigate primary care-based follow-up in a setting where patients and clinicians actually have the opportunity to experience primary care-based care as well.

The aim of the current study was, in the context of an ongoing randomized clinical trial, to analyze the reach of the trial and to identify and evaluate constructs relevant to the implementation of primary care-based prostate cancer follow-up. We used the Consolidated Framework for Implementation Research (CFIR) to guide the evaluation of the implementation of primary care-based prostate cancer follow-up [17]. 

## 2. Materials and Methods

### 2.1. Design and Study Population

This mixed-methods process evaluation investigated a primary care-based follow-up program for prostate cancer survivors in the Netherlands in an RCT setting. A detailed description of the design of the RCT has been published previously [6]. Briefly, eligible prostate cancer survivors who had completed primary treatment (prostatectomy or radiotherapy) for localized prostate cancer and without evidence of recurrence were recruited between July 2018 and September 2021. In total, 402 consenting men were randomized to either specialist- (usual care) or primary care-based (intervention group) follow-up. 

For the interviews, we recruited prostate cancer survivors (randomized to primary care-based follow-up), GPs (those who carried out the follow-up of at least one prostate cancer patient), and specialists (urologists, radiation oncologists, and physician assistants) who were participating in the PROSPEC trial for at least one year. 

**Trial Registration:** Netherlands Trial Registry, Trial NL7068 (NTR7266). Prospectively registered on 11 June 2018.

Ethical approval was obtained from the institutional review board of a comprehensive cancer center in the Netherlands (IRBd19-251). All participants signed written informed consents before participating in the study.

### 2.2. Data Collection

We used a mix of quantitative and qualitative data to describe the reach and to identify and evaluate constructs relevant to the implementation of the trial (see Table 1). We used the CFIR determinant framework to guide the evaluation of the implementation of primary care-based prostate cancer follow-up [17]. 

To describe the reach, we collected data from the research logbook of the PROSPEC trial. The reach was calculated over patients who received the information letter of the trial. In addition, we conducted semi-structured interviews with patients, GPs, and specialists. We developed an interview guide using the CFIR domains (see Table S1). Data about prostate cancer survivors’ socio-demographics (date of birth, marital status, and educational level) and clinical characteristics (date and type of treatment, and risk group) were collected as part of the RCT. GPs and specialists completed a brief questionnaire about socio-demographics (date of birth and sex) and their work situation (type of healthcare professional and type of GP practice/hospital).

### 2.3. Study Procedures

For the interviews, we used a purposive sampling strategy to include a maximum variation of patients, GPs, and specialists. Members of the research team personally invited participants. The research team informed the participants and provided information letters; written informed consent was obtained. All interviews were audio-recorded.

### 2.4. Data Analysis

For the interviews, descriptive statistics were used to characterize the study sample. The audio recordings of the interviews were transcribed verbatim. To safeguard the anonymity and confidentiality of the participants, names were removed from the transcripts. The interviews were analyzed in a systematic way by four researchers (BW, EA, SC, and AB). The transcripts were analyzed according to the procedure for thematic analysis as described by Braun and Clarke (see Table 2) [18]. Three researchers independently coded the transcripts of the interviews, using an inductive approach. We continued to recruit participants until we reached data saturation. A third author (AB), not involved in the initial theme development, was consulted to review the themes. Finally, consensus was reached and the final themes were developed (see Table S2). 

RQDA [19], R package [20] for computer-assisted qualitative data analysis, was used to perform the thematic analysis.

## 3. Results

**Figure 1:**
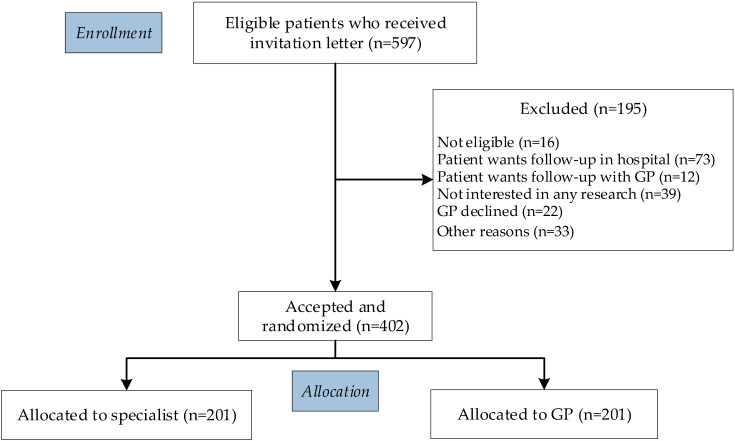
Consort flow diagram.

In total, 597 patients with localized prostate cancer from 12 hospitals (1 academic hospital, 1 comprehensive cancer center, 6 top clinical hospitals, and 4 community hospitals) across different regions in the Netherlands were invited to participate in the RCT. The reach of the study is presented in Figure 1: 16 (3%) patients were not eligible, 157 (26%) patients declined (of whom most preferred follow-up in the hospital), and 22 (4%) GPs declined to participate. Ultimately, 402 (67%) patients were randomized to specialist- (*n* = 201) or to primary care-based (*n* = 201) follow-up.

### 3.1. Interviews

In total, we interviewed 15 patients, 10 GPs, and 8 specialists (see Table 3). From the thematic analyses, we identified six overarching themes: structure of prostate cancer follow-up care, communication between primary and secondary care, clinical competencies, facilitators of and barriers to primary care-based follow-up, and organizational requirements for the implementation of primary care-based follow-up (see Table 4; including quotes to illustrate the data).

### 3.2. Structure of Prostate Cancer Follow-Up Care

The structure of prostate cancer follow-up as described by the participants aligned with the provided guideline. In both primary and secondary care, follow-up visits focused on PSA measurements. All specialists indicated that they focus primarily on physical problems and that they only discuss psychosocial problems at the patient’s request, and they usually refer patients with psychosocial problems to a nurse specialist, the GP, or a psychologist. The two radiation oncologists we interviewed indicated that they often speak with their patients who were treated with hormonal therapy about psychosocial problems (e.g., self-image and relationship issues). All GPs indicated that it was easy for them to discuss the psychosocial aspects of the cancer, but it was more difficult to discuss and manage prostate cancer-specific problems. Some of the GPs combined prostate cancer follow-up with other chronic disease care.

Almost all patients indicated having a good doctor–patient relationship. Some patients thought their follow-up appointments with the GP were less organized than what they had experienced in the hospital and some patients reported receiving more PSA measurements than described in the guideline. Two patients reported not being able to discuss prostate cancer-specific problems with their GP; one patient believed the GP could not help him and one patient struggled (in general) with requesting help. Within this theme, we observed that some patients were worried about the PSA value and therefore consulted the GP more often than the guideline prescribed. Some patients were proactive in consulting their GP, while others reported being more passive.

### 3.3. Communication between Primary and Secondary Care

None of the specialists experienced problems in the communication with primary care. All GPs indicated that the communication between primary and secondary care should be improved: not all GPs received clinical treatment information from the specialists and direct access to the specialist was perceived as difficult. GPs believed that the communication with specialists should be more accessible and transparent, preferably with one (nurse) specialist per cancer type per hospital. None of the patients was aware of any communication having taken place between primary and secondary care providers.

### 3.4. Clinical Competencies of Primary Care Follow-Up 

Specialists agreed that GPs could take primary responsibility for follow-up care, but only when there are national guidelines in place, when the PSA value is normalized, and when there are no major complications after treatment or prostate cancer-specific problems that require active treatment. Some specialists indicated that they were not sure as to whether GPs are able to manage prostate cancer-specific problems because they had not had any contact or had not received any inquiries from them. In addition, some specialists specifically mentioned that they expect the GP to be more skilled in the area of psychosocial care. 

All of the GPs believed that they are competent enough to provide prostate cancer follow-up. Some of the GPs mentioned that it was difficult to answer specific questions about their patients’ cancer prognoses and to provide follow-up care to patients who had many prostate cancer-specific problems.

Most of the patients indicated that primary care is the appropriate place for their follow-up, especially when they have few complaints. Nevertheless, most patients mentioned they would like to be referred back to the hospital if they develop more prostate cancer-related symptoms.

### 3.5. Facilitators of Primary Care-Based Follow-Up

The participants mentioned several advantages of primary care-based follow-up: the GP is closer to home, it is more accessible, it is more efficient and less expensive, GPs might combine cancer follow-up with other chronic disease management, and the hospital can focus on patients who are undergoing active treatment. Patients also mentioned several advantages of primary care: it is easier to make an appointment, the GP spends more time with them, the GP knows the patient better, and there is less emotional burden at the GP’s office, whereas the hospital environment can be emotionally upsetting.

### 3.6. Barriers to Primary Care-Based Follow-Up

Perceived barriers to primary care-based follow-up included limited knowledge and expertise among GPs. Some specialists were also concerned that patients would be lost to follow-up. Most of the GPs indicated having some issues with providing follow-up care, as it may result in an increased caseload of patients and a greater demand on capacity. The majority of the patients indicated no barriers to primary care-based follow-up, despite them having to be more proactive in consulting their GP. 

### 3.7. Organizational Requirements for the Implementation of Primary Care-Based Follow-Up

**Figure 2:**
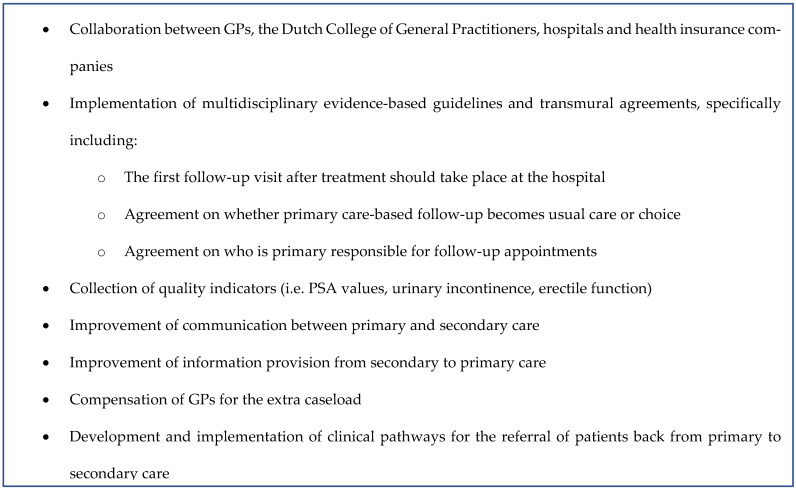
Organizational requirements necessary for the implementation of primary care-based follow-up.

Throughout the interviews, the participants mentioned several organizational requirements that have to be taken into account before implementing primary care-based follow-up. These requirements are presented in Figure 2. 

## 4. Discussion

We found that the PROSPEC trial reached 402 out of 597 prostate cancer survivors (67%). The reach of this trial was comparable to previously published RCTs investigating primary versus secondary follow-up care among patients with breast and colorectal cancer (accrual rates between 55–67%) [21,22,23]. This indicates that primary care-based follow-up is acceptable to the majority of cancer patients, and this seems to be similar across cancer types. Nevertheless, this number might be different outside of an RCT setting. 

In line with previous studies, our results also indicated that GPs discussed the psychosocial context of cancer with patients, while not all specialists reported doing so [16,24]. Besides, patients mentioned that the GP-patient relationship is more personal. A previous study among prostate cancer survivors also reported that patients rate their GP higher than oncologists in terms of interpersonal relationship [25]. A good doctor–patient relationship is very important and associated with better health outcomes, especially in longitudinal care (seeing the same doctor) and with positive consultation experiences (patients’ encounters with the doctor) [26].

Interestingly, we found that some patients were proactive in consulting their GPs for PSA measurements or problems. It is known from previous research that active participation in medical consultations increases commitment to treatment plans, results in better provision of information and support from physicians, and improves satisfaction with care [27]. If we want to implement primary care-based follow-up, it is important that future studies investigate how to support patients to ensure patients receive care according to the follow-up guideline. 

As reported by studies before, GPs also argued that a transition of oncology care to primary care would result in a greater demand on capacity [15,28]. If we want primary care to be more involved in the cancer care continuum, it is critical to address this issue of GP workload and compensation. 

Some GPs expressed a lack of confidence in managing prostate cancer-specific problems. This is in line with previous research, where GPs indicated having confidence in performing non-cancer tasks such as pain management and psychosocial support, but less confidence in surveillance testing and managing long-term effects of cancer [14,29]. Often, this uncertainty is associated with minimal training in follow-up care and a lack of evidence-based guidelines [14,28,30]. It is, therefore, important to implement (inter-)national multidisciplinary cancer follow-up care guidelines, including up-to-date disease management, for a successful implementation of primary care-based follow-up. 

Another reason for a lack of confidence in relation to providing primary care-based follow-up can be the poor communication between primary and secondary care [14,28]. GPs reported that access to specialists and, consequently, access to information is difficult. Future studies should develop and implement intervention strategies aimed at improving the communication and information provision between primary and secondary care. 

Limitations of this study include the fact that the results of the RCT on the effectiveness, adoption, and maintenance of primary care-based follow-up care are not yet available. In addition, we note that participants in the qualitative part of the current study had accepted the invitation to participate in our RCT. This, by definition, resulted in a selective sample of patients who most likely had good relationships with their GPs and were positive toward primary care-based follow-up. In addition, the patients we interviewed were relatively healthy, despite our attempts to recruit patients with a wide range of prostate cancer-related problems. Finally, we were unable to investigate attitudes toward primary care-based follow-up developed after sustained experience with it, as the patients, GPs, and specialists were experiencing it for the first time.

Strengths of this study include the use of the CFIR implementation research framework. This framework allows us to systematically evaluate a complex intervention and will contribute to a successful implementation of primary-based prostate cancer follow-up care [17]. In addition, the interviews were transcribed verbatim and analyzed by different researchers using a thematic analysis to assure the robustness of the findings. Furthermore, we aimed to include a heterogeneous population by including GPs and specialists working in different types of hospitals and practice sizes. Finally, we included both GPs who were in favor of and those who were critical toward primary care-based follow-up.

## 5. Conclusions

In conclusion, the results of this process evaluation indicate that patients, GPs, and specialists are positive toward primary care-based follow-up. Most of the participants indicated that primary care could make prostate cancer follow-up care more personal, efficient, and sustainable. If the RCT shows that primary care is equally as effective and safe as specialist-based follow-up, this process evaluation can be used to understand how the intervention is working, what challenges need to be overcome, and which requirements are necessary to enable successful implementation of prostate cancer follow-up in primary care.

## Figures and Tables

**Table 1:** Components of the process evaluation, including CFIR domains [17].

Components	Definition	Source
Reach	The number and proportion of the target population participating in this intervention	Research logbook
CFIR Domains	Definition	Source
Intervention characteristics	The characteristics of the intervention when implemented in an organization	Qualitative interview questions
Outer setting characteristics	The political and social context within which an organization resides	Qualitative interview questions
Inner setting characteristics	The structural, political and cultural context through which the implementation process will proceed	Qualitative interview questions
Individual characteristics	The knowledge, beliefs, attitudes and expectations of the individuals involved in the intervention	Qualitative interview questions
Implementation process	Processes and change that are needed for a successful implementation	Qualitative interview questions

Abbreviations: CFIR = Consolidated Framework for Implementation Research.

**Table 2:** Overview transcript analysis.

Phase	Coding method	Performed by
1. Familiarizing yourself with your data		BW, EA, SC
2. Generating initial codes	Inductive approach	BW, EA, SC
Iterative process	Consensus-based codebook	BW, EA, SC
Review	Consensus-based codebook	BW, EA, SC, AB
Data saturation	Final codebook	BW, EA, SC, AB
3. Searching for themes	Using CFIR framework	BW, EA, SC
4. Reviewing themes	Using CFIR framework	BW, EA, SC
5. Defining and naming themes	Using CFIR framework	BW, EA, SC, AB
6. Producing the report		BW, AB

**Table 3:** Characteristics of interview participants.

Demographics	Patients, *n* = 15 (%)	GPs, *n* = 10 (%)	Specialists, *n* = 8 (%)
Age at interview in years, M (SD)	67 (6)	53 (10)	47 (7)
Sex	--		
Female	--	4 (40)	0 (0)
Male	15 (100)	6 (60)	8 (100)
Marital status		--	--
Partner	14 (93)		
No partner	1 (7)		
Educational level ^a^		--	--
Low	3 (20)		
Intermediate	1 (7)		
High	11 (73)		
Clinical characteristics			
Primary treatment		--	--
Radical prostatectomy	13 (87)		
Radiotherapy	2 (13)		
ADT	1 (7)		
Time since treatment in months, M (range)	20 (17–25)	--	--
LPC risk group ^b^		--	--
Low	5 (33)		
Intermediate	5 (33)		
High	5 (33)		
Information healthcare professionals			
Type GP practice	--		--
Duo practice		5 (50)	
Group practice		5 (50)	
Type of healthcare professional	--	--	
Urologist			5 (62)
Radiation Oncologist			2 (25)
Physician Assistant			1 (13)
Type of hospital	--	--	
Academic hospital			1 (12)
Top clinical hospital			3 (38)
Comprehensive cancer center			3 (38)
Community hospital			1 (12)

Abbreviations: GP = general practitioner, ADT = androgen deprivation therapy, M = mean, SD = standard deviation, LPC = localized prostate cancer, -- = not applicable. ^a^ Educational level was classified into low (no, lower (vocational) education), intermediate (secondary vocational education), and high (higher (vocational) education and university); ^b^ LPC risk group was classified according to the EAU guidelines [3].

**Table 4:** Quotes of patients, GPs, and specialists about primary care-based prostate cancer follow-up according to the themes from the transcript analysis.

Theme	Quotes (Examples)
Structure of prostate cancer follow-up care	P3: ‘Once my wife was also very worried, and then I had my PSA checked because it does not help me if she gets nervous.’
	GP3: ‘I see this person more often for all sorts of reasons, so sometimes it happened that I just, ehm, combined it (i.e., follow-up consult) with complaints of his respiratory system or something like that.’
	S8: ‘We also offer people a psychologist or social worker, if there is a need. But the physical and oncological examination are the main aspects.’
Communication between primary and secondary care	P1: ‘No, I did not experience any of that (i.e., communication).’
	GP9: ‘In general, it is always difficult to reach a specialist, or you will be called back but not at the moment the patient is with you.’
	S6: ‘No, I have never heard anything from the GPs. That shows how redundant we really are, at least for this part (i.e., follow-up).’
Clinical competencies of primary care-based follow-up	P4: ‘What the GP did well, I must say, was covering everything…like, how is it going physically, how is it going psychologically, do you have specific questions at a physical level, about urinary incontinence or erectile dysfunction, or are you tired, or do you still have…?’
	GP8: ‘Especially information about prognosis, what are the chances that things can come back, I cannot of course, 1,2,3, I do not have those numbers ready of course, no.’
	S2: ‘I have actually had no feedback from GPs who said, ‘’Hey, I have a patient here with erectile problems and I am not sure what to do.” Or you (i.e., study team) provide GPs with excellent information about this, or they do not have questions, or they do not call us. I am not quite sure.’
Facilitators of primary care-based follow-up	P5: ‘And compared to the hospital, you know… Emotionally that is better. Better to do this (i.e., follow-up) with your GP. And when that is an option, then that is very positive.’
	GP5: ‘Well, I think it is very patient-friendly when he does not have to go to the hospital, it will save costs, the effort for me is little, and it is also pleasant for me that I can speak twice a year to someone who had prostate cancer.’
	S3: ‘It really results in extra time in which you can take care of people with bigger problems, who really need the hospital setting.’
Barriers to primary care-based follow-up	P13: ‘Yes, with the GP you have to undertake action yourself. That is, you know, a GP does not have a system to call people, so if you have complaints you have to go to the GP yourself.’
	GP1: ‘The disadvantage is that we do not get one extra penny for it. But I do believe that, uh, primary care is capable of doing this. But then we kind of need… or then we should receive compensation or extra staffing.’
	S1: ‘That is my fear you know, that they (i.e., GPs) will not refer them (i.e., patients) back. Or that they are too late, or not frequently measure their PSA. And then we will lose the window of curability.’
Organizational requirement for the implementation	See text

Abbreviations: P = patient, GP = general practitioner, S = specialist.

## Data Availability

The dataset used and analyzed during the current study will be available from the corresponding authors (stored in a data repository at the Netherlands Cancer Institute) on reasonable request.

## Supplementary Materials

The following supporting information can be downloaded at https://www.mdpi.com/article/10.3390/cancers14133166/s1, Table S1: Interview guide based on CFIR domains (18); Table S2: Themes mapped to the CFIR framework.
