# Supplementary material for: Specialist versus Primary Care Prostate Cancer Follow-Up: A Process Evaluation of a Randomized Controlled Trial
Source: Cancers (Basel). 2022 Jun 28;14(13):3166. doi: 10.3390/cancers14133166 (PMC9264897; doi:10.3390/cancers14133166)
Supplement: Supplementary file 1 [file cancers-14-03166-s001.zip › Supplementary Table S1.pdf]

Supplement Table S1. Interview guide based on CFIR domains (18)

CFIR domains	Stakeholder	Main interview questions
**Intervention characteristics**		
Complexity and difficulty of implementing PROSPEC in everyday practice	GPs and specialists	What are your experiences with implementing primary care-based follow-up care for prostate cancer patients in GP offices?
	GPs	How long does a follow-up consultation take? Did you have enough time to complete a follow-up consultation?
	GPs	What are your experiences with performing periodic PSA measurements? What are your experiences with the logistics for PSA measurements (face-to-face or by telephone)?
Perception of quality and validity of evidence supporting primary care-based follow-up care	GPs and specialists	Did you make use of the follow-up guideline?<br>What is your opinion on the follow-up guideline? What are your experiences with the follow-up guideline?
	Patients	What did you discuss with your GP/specialist during a follow-up consultation? Were you able to talk about prostate cancer specific or psychological problems? Did you discuss other problems, besides prostate specific problems?
	GPs and specialists	What did you discuss during a follow-up consultation? Did you discuss prostate cancer specific or psychological problems? What are your experiences with discussing prostate cancer specific-problems and psychological problems?
	GPs	Are you combining prostate cancer follow-up care with other (chronic) care?
Relative advantage of the PROSPEC	All	What do you see as a relative advantage of primary care-based follow-up care versus an alternative solution? What do you see as a disadvantage of primary care-based follow-up care?
**Outer setting characteristics**		
Communication between hospitals and GP	GPs and specialists	What is the quality and nature of communication between hospitals and GPs?
	Patients	How do you perceive the communication between the hospital and your GP?
External policies and peer pressure	GPs and specialists	Do you perceive external pressure to perform primary care-based follow-up care?
**Inner setting characteristics**		
Ability to implement	GPs	Would GP practices be able to implement prostate cancer follow-up care?
Available resources	GPs	Are there enough resources available? (i.e. time, room, equipment, or do you expect reimbursement)
Organizational issues	GPs	Are there any organizational issues?
Self-management	Patients and GPs	Who was responsible for the follow-up care appointments? How did you perceive that?
Access to knowledge and information	Patients and GPs	Did you have enough access to information about prostate cancer follow-up and aftercare?
**Individual characteristics**		
Knowledge and beliefs	All	How do you perceive the knowledge and skills of GPs for prostate cancer follow-up care?
What are the attitudes of the key stakeholders	All	Do you think the follow-care can take place at the GP?

Participation and expectations	All	What was your reason to participate in the PROSPEC trial?
	All	What are/were your expectations of the PROSPEC trial?
	All	Do you plan to continue primary care-based follow-up care after the trial?
**Implementation process**		
Views and recommendations for follow-up and aftercare of prostate cancer survivors	All	What key features of the intervention should be included or removed for optimal effect and implementation?
	Specialists and GPs	What are barriers and facilitators for implementing primary care-based follow-up care?
	All	Would you like to add anything we did not discuss today?
