# Supplementary material for: Specialist versus Primary Care Prostate Cancer Follow-Up: A Process Evaluation of a Randomized Controlled Trial
Source: Cancers (Basel). 2022 Jun 28;14(13):3166. doi: 10.3390/cancers14133166 (PMC9264897; doi:10.3390/cancers14133166)
Supplement: Supplementary file 1 [file cancers-14-03166-s001.zip › Supplementary Table S2.pdf]

Supplement Table S2. Themes mapped to the CFIR framework

CFIR domains	CFIR constructs	Themes					
		**Structure of follow-up**	**Communication**	**Clinical competencies**	**Facilitators**	**Barriers**	**Organizational requirements**
**Intervention characteristics**							
	Intervention Source	X					
	Evidence Strength & Quality	X		X			
	Relative advantage			X	X	X	X
	Adaptability			X			X
	Complexity			X		X	X
	Design Quality and Packaging	X			X		
**Outer setting**							
	Patient Needs & Resources	X			X	X	
	Cosmopolitanism		X				
	External Policies & Incentives		X				
**Inner setting**							
	Networks & Communications		X				
	Culture	X	X				X
	Implementation Climate	X				X	X
	Readiness for Implementation	X					X
**Characteristics of individual**							
	Knowledge & Beliefs about the intervention	X		X		X	X
	Self-efficacy	X		X			
	Individual Stage of Change			X	X		
	Individual Identification with Organization	X			X	X	
	Other Personal Attributes	X					
**Process**							

	Planning				X
	Engaging				X
	Executing	X			
	Reflecting & Evaluating		X	X	X

Note: The following CFIR constructs did not appear in the themes of were not yet discussed and will be analyzed and published when the 2-year follow-up of the RCT has ended: trialability, cost, peer pressure, and structural characteristics.
